# Breastfeeding Awareness and Empowerment (BAE): A Black Women-Led Approach to Promoting a Multigenerational Culture of Health

**DOI:** 10.3390/soc12010028

**Published:** 2022-02-18

**Authors:** Rebecca Duncan, Jabina Coleman, Sharon Herring, Meg Kawan, Christy Santoro, Meghana Atre, Aleigha Mason, Shawana Moore, Aparna Kumar

**Affiliations:** 1 Breastfeeding Awareness and Empowerment (BAE), Philadelphia, PA 19132, USA; 2 Obstetrics and Reproductive Sciences, Temple University, Philadelphia, PA 19140, USA; 3 Children’s Hospital of Philadelphia (CHOP) Karabots Center, Philadelphia, PA 19139, USA; 4 Mailman School of Public Health, Columbia University, New York, NY 10032, USA; 5 School of Nursing, University of Pennsylvania, Philadelphia, PA 19104, USA; 6 College of Nursing, Thomas Jefferson University, Philadelphia, PA 19107, USA

**Keywords:** lactation, breastfeeding, perinatal mental health, reproductive justice, trauma-informed care, strength-based, community-based, black maternal health, health equity, disparities

## Abstract

**(1) Background:**

Critical gaps in the U.S. healthcare system perpetuate adverse reproductive health outcomes for Black people. Grounded in reproductive justice and trauma-informed care, Breastfeeding Awareness and Empowerment (BAE) has developed a program titled BAE Cafe to directly address these gaps by providing community-based lactation and perinatal mental health support. A literature review identified key programmatic gaps, namely, access to knowledge relevant to troubleshooting breastfeeding, peer support, community support and healthcare system support, and system-level factors that impede families and communities from accessing lactation support.

**(2) Methods:**

This paper describes BAE Cafe through a group process observation and participant survey.

**(3) Results:**

The observation of groups highlighted the core elements of the BAE Cafe model: knowledge, support and mental health support in a peer driven format. Participant survey feedback was overwhelmingly positive and highlighted the critical importance of lactation support for Black women by Black women and BAE’s role in participants’ decisions to continue breastfeeding.

**(4) Conclusions:**

BAE Cafe is a replicable, scalable, peer-driven and low-barrier intervention that has the potential to improve outcomes for Black families. Additional research and investment are now needed to assess large-scale implementation to reduce disparities and address health inequity across different contexts and settings.

## 1. Introduction

Healthy People objectives set forth by the U.S. Department of Health and Human Services have included targeted breastfeeding goals as a means of disease prevention and health promotion since the early 2000s [1]. After years of continuing to miss the mark, the Surgeon General formally recognized the significance of this public health crisis in 2011 and released a national call to action to address the prevailing needs [2]. Public health experts also recognized persistent racial disparities in breastfeeding rates, and reported the multiplicative implications for both maternal and early childhood health outcomes that widen across the lifespan [3,4]. Nonetheless, despite the wealth of supporting evidence for the imperative recommendations made by the World Health Organization and the American Academy of Pediatrics regarding breastfeeding and human milk as optimal first food, breastfeeding rates for initiation and continuation among Black childbearing families across the United States continue to be significantly low when compared to national statistics for all groups [5,6].

The Centers for Disease Control National Immunization Survey data from 2011 to 2015 revealed that for Black infants, breastfeeding initiation rates were significantly lower in 23 states, with 14 states having differences of more than 15 percentage points. Black infants were also 10 percentage points less likely than White infants to be exclusively breastfed at 6 months in 12 states and at 12 months in 22 states [5]. This disparity is locally reflected in Philadelphia data. According to the 2017 Community Health Assessment published by the Philadelphia Department of Public Health, the initiation rate for breastfeeding among Black infants was up to 10.9 percentage points lower than those belonging to other racial and ethnic groups [7]. There is an urgent need for comprehensive interventions that effectively increase the initiation and continuation of breastfeeding among Black childbearing families in the United States.

Breastfeeding Awareness and Empowerment [BAE] [8] was formed in direct response to this need. Combining lived experience, years of professional expertise and guidance from the Black Mamas Matter Alliance’s call to action to address disparities in perinatal health outcomes for Black families, BAE aims to protect, promote and uplift black breastfeeding [9]. By raising awareness and creating spaces for breastfeeding empowerment, BAE seeks to eliminate disparities and promote a lasting, multigenerational culture of health.

### 1.1. BAE Addresses Racial and Ethnic Breastfeeding Disparities

In recent years, national attention has increased on the critical gaps in the United States health care system that have failed to address systemic racism and disproportionately perpetuate preventable, adverse reproductive health outcomes for Black people [10,11]. While this call to action has largely focused on maternal and infant morbidity and mortality, breastfeeding and human milk as first food is also an imperative health indicator that has short- and long-term health implications for both birthing people and babies [12]. Emerging research affirms the pervasiveness of these inequities, which builds on the work of reproductive justice advocates who have long named the impact of systemic racism in shaping perinatal outcomes [13]. Research reveals that gaps to eliminating racial disparities in Black breastfeeding outcomes include: failure to address disparities in breastfeeding rates for Black childbearing families [14,15]; a lack of representation and visibility of lactation professionals of color [16–18]; a lack of community-based lactation resources that integrate reproductive justice and trauma-informed care [19,20]; and a lack of comprehensive lactation support models that incorporate perinatal mental health [21,22].

Stemming from over 25 years of experience serving childbearing families through public health, clinical services and community organizing, BAE embodies the work of Jabina Coleman LSW, MSW, IBCLC, a Licensed Social Worker and International Board Certified Lactation Consultant and Rebecca Duncan RN, BSN, IBCLC, Doula, a Public Health Registered Nurse, International Board Certified Lactation Consultant and community doula. Co-created by two Black perinatal health professionals who have spent years working on the frontlines of perinatal care for systematically disenfranchised families throughout greater Philadelphia, this collaboration seemed inevitable as their work continued to intersect at critical points in their clients’ lives. Understanding the value of interprofessional collaboration through their working relationship, they began partnering in 2016 to develop content and grassroots programming to uniquely address the unmet needs of their community.

This local grassroots work started in response to their experience and the surmounting public outcry surrounding adverse perinatal health outcomes for Black families in the United States. With the aim of protecting, promoting and uplifting Black breastfeeding, BAE was birthed through the integration of reproductive justice principles [23] and trauma-informed care practices [24] into programming that centers systematically marginalized communities. BAE also specifically addresses perinatal mental health, a key component of perinatal health that is often treated separately and left unaddressed by current breast-feeding support models and the current healthcare system [25]. Lastly, BAE operates under a for us, by us model through an emphasis on racially concordant care and shared lived experiences between care facilitators and Black families.

Breastfeeding Awareness and Empowerment (BAE) includes four programmatic components: BAE Cafe, Tools for BAE, BBQ4BAE and BAE Insight. BAE Cafe is a unique support group model for comprehensive, community-based lactation support. Tools for BAE is a curriculum to train a diverse cross-section of organizations, professionals and individuals to provide comprehensive lactation support for childbearing families. BBQ4BAE is an annual, family-centered cookout to celebrate Black Breastfeeding Week [26] in the community. BAE Insight is a consultation service to advise organizations looking to implement this unique approach of comprehensive, community-based lactation support. Each component integrates reproductive justice and trauma-informed care to center marginalized communities and equip participants with knowledge, tools and resources to provide support that addresses client-centered needs. For the purpose of this paper, we focus on the remarkable success of BAE Cafe.

### 1.2. BAE Cafe Employs an Innovative and Evidence-Based Approach to Targeting Gaps in Breastfeeding Education, Instruction and Support for Black Families

BAE Cafe is one of four programmatic components of BAE. In addition to integrating reproductive justice, trauma-informed care and perinatal mental health, this unique support group model specifically honors participants’ ability to make the best decisions to nourish their families. This strengths-based practice approach to supporting positive behavioral change aligns with reproductive justice principles and has been identified as an essential philosophical shift from historical trends in community-based work [13,27]. Additional innovative, evidence-based program elements of BAE Cafe include racially concordant care and an emphasis on holding space during the fourth trimester to support the transition into parenthood. BAE Cafe meetings consist of regularly scheduled sessions with a trained BAE facilitator. Participants are invited to share breastfeeding successes and challenges. Group discussions include breastfeeding, the holistic discussion of perinatal mood changes and transition to parenthood. Community referral sources to trusted community partners are available for people who require a higher level of care.

Beyond evidence-based approaches to care, BAE Cafe deeply invests in trusted community partnerships to build a culture of health. With the support of pediatric providers, funding was secured to develop a streamlined referral process with a local pediatric clinic. Groups were initially hosted in collaboration with the Philadelphia Free Library and successfully expanded from one to three library sites, where BAE facilitated staff training and designated family friendly spaces for meetings. In response to the COVID-19 pandemic, BAE Cafe quickly adapted to safety guidelines and shifted to weekly virtual meetings. Outreach methods have continued to include sharing with established community partners and health providers, social media and word of mouth. Word about this program spread quickly throughout the Philadelphia community, and meetings have remained consistently well attended by 9–10 mother–infant dyads at each session. Participant feedback anecdotally highlights the value of featuring intermittent program topics of interest, accessibility of location in the community and collectively establishing meeting times that work for most people.

Lastly, BAE Cafe emphasizes community capacity building through opportunities for workforce and professional development as mechanisms for systemic change. With support from the Philadelphia Department of Public Health Division of Maternal, Child & Family Health and other local collaborators, BAE has secured funding to invest in a self-generating cycle of support. BAE Ambassadors are BAE Cafe participants who have assumed an active leadership role to promote, protect and uplift breastfeeding in the community and to advance the mission/vision of BAE. They represent BAE Cafe at local events and are a core group of participants who share collective leadership roles in exchange for an honorarium. BAE Cafe is also a gateway to diversifying the perinatal workforce and serves the dual purpose of supporting Black IBCLC candidates to complete the certification pathway through mentorship, access to training and opportunities to obtain clinical hours.

BAE seeks to share this model for dissemination to communities most at risk. In this paper, we seek to (1) review the current literature examining interventions for Black families around breastfeeding initiation, continuation and mental health support; (2) describe the key elements of the BAE Cafe model and its grounding in reproductive justice. There are few models like BAE Cafe that specifically employ strengths-based practices and draw on community partnerships to sustain health changes, but the success that BAE Cafe has shown demonstrates that parents could benefit greatly from programs based on the BAE Cafe model.

### 1.3. Evidence from Other Models on Breastfeeding Support for Black Parents

To contextualize BAE Cafe as well as identify key gaps in current models, a brief, integrative review of the literature was completed. An online search was conducted using PubMed for the years 2010–2021. The search query included keywords such as “breastfeeding”, “breast feeding”, “chest feeding”, “mental health”, “self-help groups”, “lactation support”, “lactation consult” and “African American.” The search contained Boolean operators such as “And” and “OR”. Mesh terms, which represented broader categories, were also included in the search. The primary search used yielded 159 articles. A total of 22 studies were included in the final review, 16 of which are included below. These studies were included based on a program and study description and their relevance to the scope and context of BAE’s work. The studies are summarized below and grouped by theme and content with relevance to BAE Cafe. The primary topics of the studies reviewed were: individual knowledge and beliefs, support and community/system factors. Only one study evaluated a program similar in goals and conceptual factors to BAE Cafe.

### 1.4. Individual Knowledge/Belief

Knowledge gaps are often cited as one of the many drivers leading to low breastfeeding initiation rates [2]. Among participants in the four studies examined, new parents—including Black parents—demonstrated an understanding of the positive health of breastfeeding benefits (such as decreased risk of chronic diseases) for the parent and infant. A lack of knowledge of the benefits of breastfeeding was not suggested by the literature. For example, a mixed methods study by Kadakia et al. (2015) that examined the influence of bedsharing on breastfeeding found no relationship between the two factors. The hypothesis of the study was that parents who had bedsharing arrangements were more likely to breastfeed; instead, it was found that parents had a high knowledge of breastfeeding and that factors such as bedsharing were not influential on the decision to breastfeed. Rather, participants cited barriers to breastfeeding, such as breast pain, having little support and general maternal skepticism about the benefits of breastfeeding. This was true for groups with low and high socioeconomic status [28].

In a study by Johnson et al. (2015), focus groups of 29 pregnant or lactating people and 9 health professionals were engaged to better understand breastfeeding initiation and duration among African American mothers. The study found that knowledge was high around the benefits of breastfeeding. However, significant factors influencing continuation were: encountering challenges in feeding and not having support to navigate them; mental health challenges, including depression; lack of support from healthcare professionals to navigate challenges; the need for different models of breastfeeding support in the hospital and community; and the lack of culturally relevant support [29].

Lastly, Lewkowitz et al. (2018) examined secondary data from a parent intervention that assessed breastfeeding support for African American mothers in a weight management program. Survey data noted that program participants consistently ranked inconvenience, formula superiority, difficulty latching and low milk supply as primary reasons for breast-feeding cessation [30].

Taken together, the results of these four studies suggest that it is not a gap in understanding the benefit of breastfeeding to the parent and baby that is driving adverse outcomes. Rather, it is a lack of ability to navigate breastfeeding challenges as well as support to ask questions and/or gain additional knowledge that may be lacking.

### 1.5. Supports

While support from peers, communities, organizations and healthcare organizations are seen as central to the support of the breastfeeding person, four articles reviewed raised themes of social networks, close family members and trusted community members as key people involved in breastfeeding decision making [31]. A qualitative study by Carlin et al. (2019) including 28 Caucasian and African American mothers examined social networks as an influencing factor on breastfeeding. Among African American mothers, it was found that if breastfeeding was not common in their social network, they could still choose to breastfeed if one member in their network was supportive. Close family members were especially important including mothers, sisters and partners. Social norms, such as acceptability of breastfeeding in the workplace, also emerged as important. Participants also expressed that social network support can influence breastfeeding behavior and even contribute to resistance against opposing norms [32].

Intervention programs can be characterized as support. They are often high intensity and short in duration, while other forms of support may be more long lasting. In one randomized controlled trial of 328 mothers enrolled in the WIC Program, it was found that people who received a breastfeeding support intervention (BST) with phone support; home visits; and on-call access at 6, 12 and 24 weeks postpartum were more likely to breast feed at 6 weeks postpartum [33]. However, factors related to the success of interventions did not necessarily relate directly to the program but also to life factors, such as return to work, which could not be mitigated by the intervention. Another intervention among WIC Program participants found that peer support, combined with motivational video tapes, increased the likelihood of breastfeeding at 8 and 16 weeks postpartum compared to the non-intervention group; however, both groups had lower rates of breastfeeding (although less so) after 16 weeks. Factors negatively influencing breastfeeding cessation included: return to work, age and having a male infant. People with prior breastfeeding experience were more likely to continue breastfeeding [34]. Similarly, Reno et al. (2018) conducted a qualitative study of 21 African American women and three lactation peer specialists who participated in model building sessions. Three major factors were found to influence barriers and facilitators to breastfeeding. These included: return to work or school, baseline and follow-up knowledge, support, determination to continue breastfeeding and social acceptance within their group for breastfeeding [35].

A final theme that emerged is that who and how support happens matters. Robinson et al. (2019) examined the importance of this model through Facebook communities. The authors conducted a prospective cross-sectional study with online focus groups with 22 focus group participants. It examined engaging in focus groups as a meaningful way for people to receive and interact in a participatory way for breastfeeding support. This was especially true for African American mothers who identified with groups of people who had similar expressed and named identities. Regarding support, several key themes emerged in the literature: the importance of peer support, the value of shared lived experience, the importance of targeted support for the community and the value of information from other African American mothers. In addition, the experience of having a group by and for African American women as a way to support one another was critical to the intention to breastfeed and breastfeeding longevity [36].

### 1.6. Community/System Factors

A critical theme that emerged in the additional 8 studies reviewed was the importance of clinician trust, community supports and system-level factors that are supportive of breastfeeding [31]. Clinician bias, clinician lack of awareness of supports and difficulty accessing full treatments and services can contribute to the lack of initiation of breastfeeding [37]. For example, in a qualitative study by Lutenbacher et al. (2016) with focus groups of 16 Black women, the most important theme around breastfeeding was a lack of support from healthcare providers or systems. The most influential factor was family support and seeking information from the internet around breastfeeding. The authors conclude that having “diverse, culturally sensitive, and user-friendly options” are critical for supporting breastfeeding among Black women [38]. Another qualitative study by Johnson et al. (2015) of 8 pregnant African American women, 21 new mothers and 9 lactation support specialists highlighted that paid leave and/or workplace policies that support breastfeeding are critical to maintaining breastfeeding. Participants also expressed the importance of training for health care professionals, follow through on workplace standards and regulations and peer-led support for breastfeeding [39].

In a study by Obeng et al. (2015) of two focus groups (*n* = 20) conducted by the Indiana Black Breastfeeding Coalition (IBBC), participants cited healthcare systems; lactation support; and the benefits of breastfeeding, such as health benefits and bonding, as positive. However, they noted that negative perceptions by family and friends, lack of information from healthcare providers and unanticipated challenges in their breastfeeding journey contributed to stopping breastfeeding [40]. In a qualitative study by Cottrell et al. (2013) of 253 African American women aged 18–35 in Florida, people reported receiving limited breastfeeding information. Most women chose breastfeeding due to the potential benefits, clinician support and attending a breastfeeding class or other support group. Support at the time of birth, latch assistance and breast pump availability also helped women to initiate breastfeeding. Supplementation and cessation were related to latch issues; pain; and concerns of supply, mother–infant separation and medical complications. However, women who did not choose breastfeeding cited concerns of pain, time constraint or return to work/school, or felt uncomfortable with breastfeeding. This study highlights the importance of supports at the time of birth and afterwards to support breastfeeding initiation and duration [41]. Similarly, an analysis of maternal narratives among African American and African mothers by Fabiyi et al. (2016) notes that African American mothers were more likely to report dissuasive attitudes, ambivalence around breastfeeding, lack of family support and return to work as reasons for discontinuing breastfeeding and supplementing or replacing it with formula feeding. Particularly salient was the impact of healthcare professionals on the decision to breastfeed or not. Clinician interaction was a key feature of maternal narrative [42].

A final study by Lewallen et al. (2010) of African American women ages 18 and older who were currently breastfeeding examined barriers and facilitators to breastfeeding. Themes elicited included a lack of information about breastfeeding, difficulties with return to work or feeding in public and a lack of sustained support for breastfeeding. Participants expressed that having more information during the prenatal period, sustained breastfeeding assistance and support from people of color would be beneficial in helping them sustain breastfeeding over time [43]. Of note, one study by Fischer et al. (2014) suggests that there may be cultural factors that increase the acceptability of formula feeding as a response to breastfeeding challenges. The study among 42 African American mothers versus other self-identified groups of mothers highlights that system-level issues, such as provider bias, discomfort with reaching out to clinicians and difficulty finding support, as well as poor workplace accommodations, may drive formula feeding rather than cultural factors or preference alone [44].

### 1.7. Programs like BAE

One program presented in the literature with goals similar to BAE exists and engages peers, champions and partners with community organizations. The initiative Avondale Moms Empowered to Nurse (AMEN) launched a “Peer-to-Peer support group” program, engaged African American mothers and evaluated the program employing community-based participatory research to better understand feeding intention and practices, as well as participation. The program has had over 110 participants as well as many support people, guest speakers and community organizations in attendance. Participant feedback cites the sense of group support, education, navigating challenges and connection to others as positive aspects of the group program. In addition, breastfeeding initiation rates in the initial neighborhood increased by 12%, from 44% to 56%. Finally, the group recognizes the impacts of racism and uplifts women of color to lead in their communities, as well as share their own lived experience [45]. The AMEN program highlights that comprehensive breastfeeding support is needed to address cited gaps, including education for troubleshooting in breastfeeding, group support, peer support, community specific supports, racially concordant care, long term follow up and connection as key programmatic elements. However, system-level factors, such as workplace return and provider bias, may not be directly part of the program as they are in BAE.

## 2. Materials and Methods

In order to describe BAE Cafe from the observer and participant perspective, group observation and a brief participant survey were conducted in the fall of 2020 in a collaboration between the BAE team and researchers from the Jefferson College of Nursing. The study was exempt from review on 22 December 2020 at Jefferson’s Institutional Review Board (IRB) because no health information was collected, and all information was de-identified. Study methods included the observation of the BAE groups by Dr. Shawana Moore, DNP, CRNP, WHNP-BC (S.M.) and Aparna Kumar, PhD, CRNP, PMHNP-BC (A.K.), researchers from the Jefferson College of nursing. S.M. and A.K. observed 4 sessions in the spring of 2021 to better understand the group process. Notes were taken and de-identified. No recordings were taken, and all participants agreed to allow S.M. and A.K. to observe sessions. A survey was also anonymously distributed in December of 2020 to better understand the participant experience of BAE (Appendix A).

## 3. Results

### 3.1. BAE Cafe Group Observation

**Figure 1.**
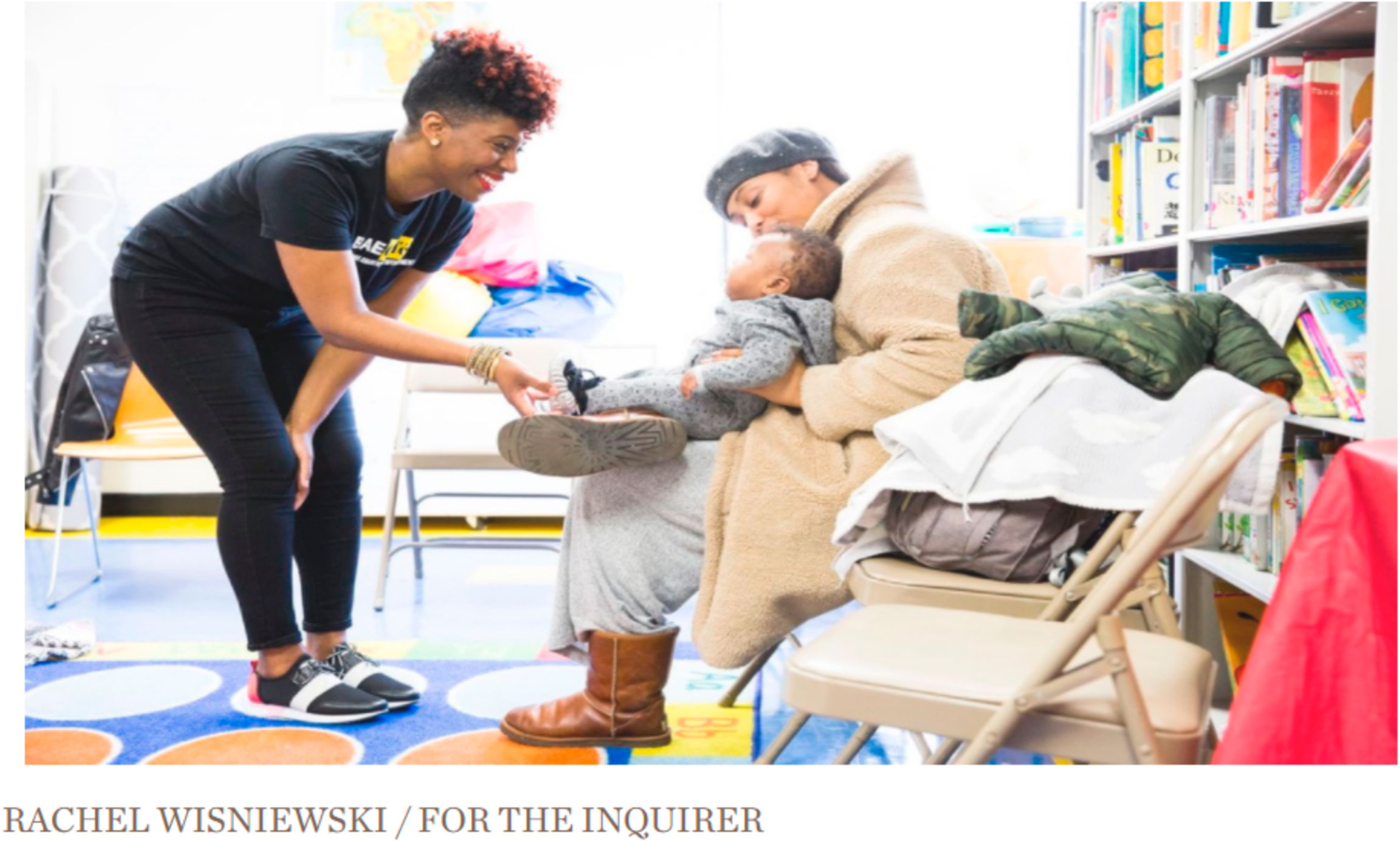
BAE Cafe feature from CHOP Policy Lab on decreasing disparities.

S.M. and A.K., researchers from Jefferson University, observed four sessions of BAE Cafe in order to understand key programmatic elements, attendee experience and interaction between facilitator and attendees (Figure 1). Key programmatic elements that emerged were: safe space, peer support and attendee directed knowledge generation. Highlights of the attendee experience are shown in the survey below. During group sessions, attendees were overwhelmingly positive and expressed feelings of support, safety and respect within a group lactation support model. Finally, the interactions of the facilitators and attendees were documented and noted to be attendee guided, centered around respect for the knowledge of the attendee and supportive to encourage the attendee to share knowledge with other group members. Based on these observations and the key elements and themes observed, a description of the groups is provided below.

**Figure 2.**
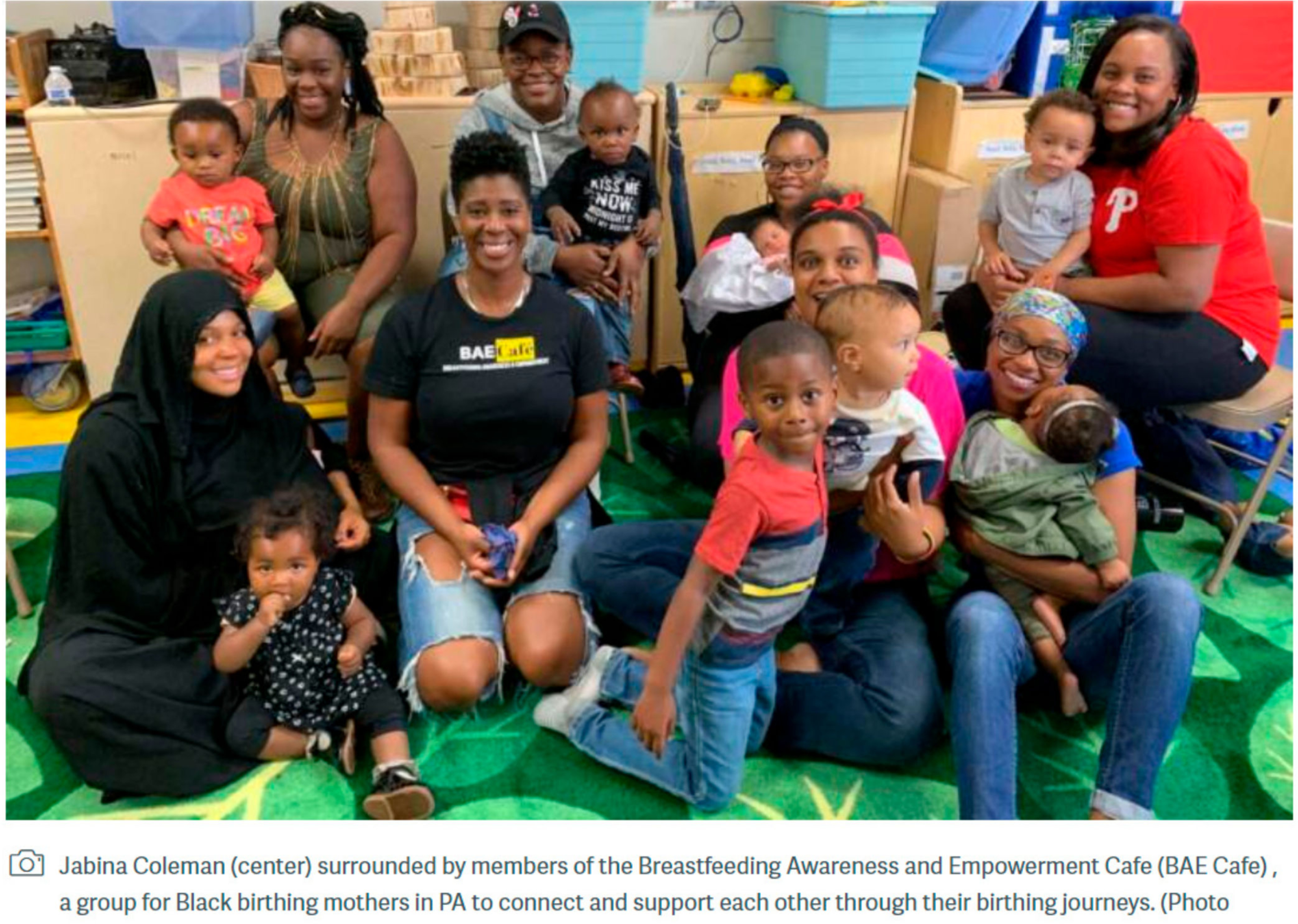
BAE Cafe feature from WHYY Philadelphia about decreasing racial gaps in breastfeeding.

BAE Cafe is a participant knowledge-driven model that encourages birthing people to find their own parenting journey (Figure 2). It is rooted in principles of reproductive justice, trauma-informed care and perinatal mental health support. This framework provides a unique support group model for comprehensive, community-based lactation support. BAE Cafe begins with an opening, a call for all members to check in. It is usually a simple check in and/or engages individuals to create space and a soft landing to start the group. This also allows the creation of a safer space. Ground rules are laid out in terms of confidentiality, as well as focus on how the process and flow of the group will be facilitated. Introductions are generally brief and focus on the participants.

From this check in of both the parent and the infant, topics are generated and the BAE facilitator discusses and engages participants to explore questions about particular topics. Topics include areas such as: first foods, family relationships, sleep, baby-led weaning, mixed feeding, chest feeding and where to go for support. Materials, supplies, tools and resources are often discussed as well. Finally, return to work and the integration of the new parenting role are often discussed. Knowledge is drawn not only from the facilitator but also from other participants in the group; in this way, each person’s experience and knowledge are valued. For instance, one participant may share their feeding strategies that were successful or not. The facilitator will discuss topics or support as appropriate while also sharing links and strategies. This is highlighted in the following scenario: when discussing protein intake for a participant’s child, the options for protein outside of traditional protein sources (animal-based or other) were discussed. Finally, mental wellness and what people are doing for themselves is also integrated. At times, specialists or experts are brought in to share knowledge and expertise. This includes topics such as: infant CPR, car seat safety, infant massage and early literacy. Each member supports one another, and a community is created. Members often stay longer than the allotted time, as well as engage with the group for one to two years into the postpartum period. One of the key components is that the community is based on mutual understanding and shared values. While people in the group represent a myriad of different identities, they all identify as people of color. However, when specialists join for special events, they are also invited to engage in the group and are included. For example, an early literacy specialist attended the group several times; although she did not identify as a woman of color, she was still included in the group.

### 3.2. Data from BAE Survey

**Figure 3.**
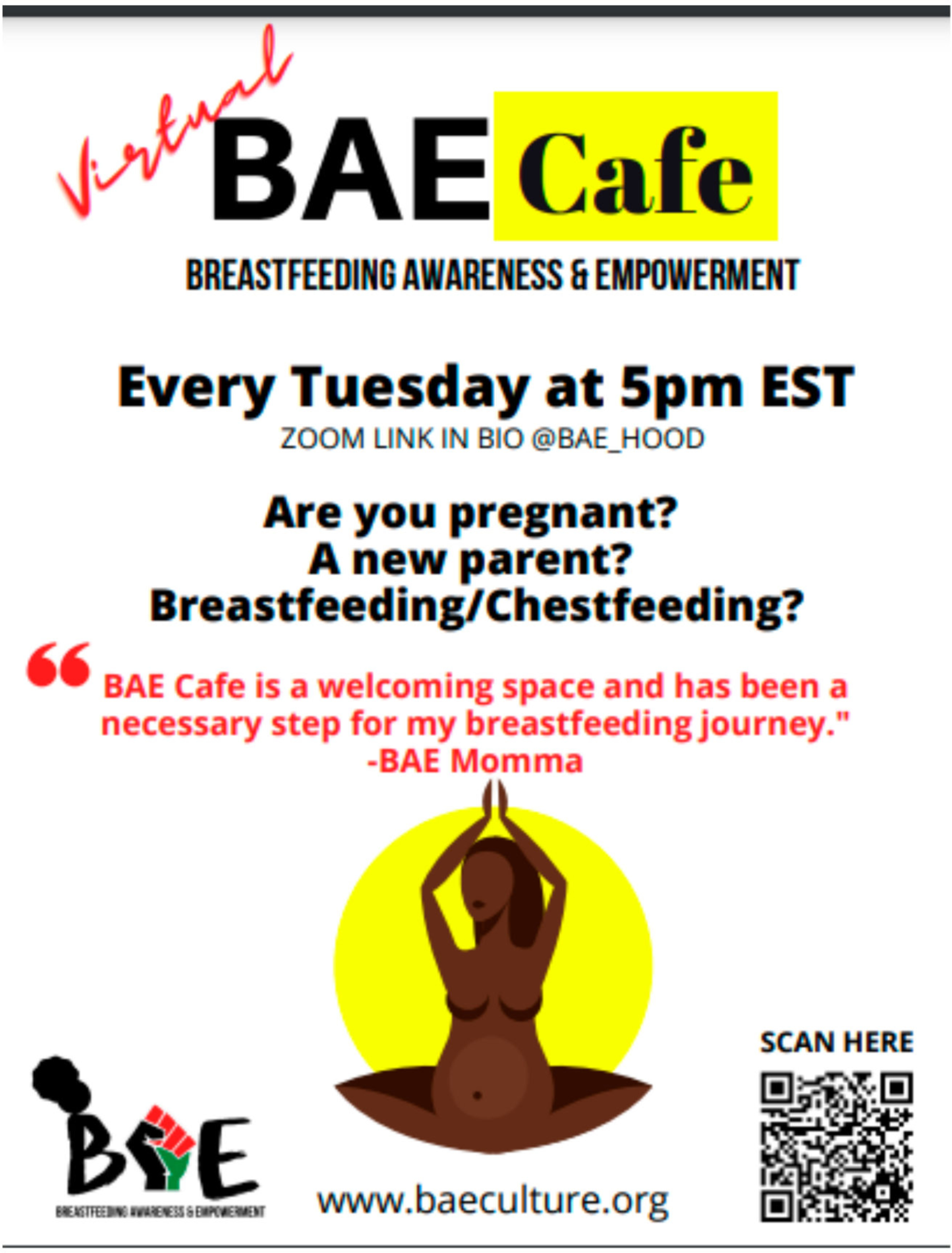
BAE Cafe virtual group flyer.

Since its inception in October 2018, BAE Cafe has reached approximately 200 mother–infant dyads in Philadelphia with significant effectiveness in improving breastfeeding initiation rates compared to national statistics in Black women (100% vs. 75%, *p* < 0.00001) and in maintaining breastfeeding for at least one year postpartum (76% vs. 30%, *p* < 0.00001), based on information drawn from a survey of BAE Cafe participants. The survey was shared within a BAE Cafe session and emailed out to 100 current and past participants. Thirty-two BAE Cafe participants responded. Survey demographics revealed that the vast majority of participants identified as Black or African American (89%) with a range of education levels (50% with a bachelor’s degree, 19% with a master’s degree and 8% with a doctoral degree), ages (24 to 46 years) and level of parity represented (50% with one child, 31% with two children, 19% with three or more children). Most participants first joined BAE Cafe in the postpartum period. However, over one-third participated while pregnant. Engagement was high, with 50% reporting attendance at more than five BAE Cafe sessions. Over one-third of participants reported that their prenatal care provider referred them to the program. Other common referral sources were social media, friends or posting at community-based settings (e.g., The Free Library of Philadelphia) (Figure 3).

The overwhelming majority of participants strongly agreed that attending BAE Cafe helped them meet their breastfeeding/chest feeding goals (96%), positively impacted their perinatal mental and emotional health (85%) and strongly agreed that BAE Cafe impacted their ability to build a supportive community of parents (77%). Participants have shared how meaningful and effective BAE has been to their own parenting journey. One participant stated “spiritually, mentally, physically and especially emotionally, BAE Cafe has made me feel great, as a human, mother, and woman”. Further validating the effectiveness of BAE Cafe, one participant stated “the welcoming space and sharing from other mothers was a very necessary step for my breastfeeding journey, and “I wouldn’t have breastfed as long as I did without it”. Aligned with this positive qualitative feedback, over 90% of participant respondents reported that they have referred other pregnant and postpartum people to BAE Cafe. Providers have routinely expressed gratitude for BAE’s programming. One pediatric provider shared:

When I first started at Children’s Hospital of Philadelphia, five years ago, I researched community breastfeeding support as I know how incredibly impactful support groups can be for breastfeeding mothers. I compiled a list of breast-feeding resources and supports for office staff to distribute to our breastfeeding families. A few months later, I was approached by a nurse practitioner who asked me for a referral. I shared an option that in my opinion was the most active, and most accessible option to families from our office. She looked at me and said, ‘But is my patient going to be the only Black woman there?’ I sighed, and answered honestly, acknowledging that there was a reasonable chance that would be the case. That conversation has stood out in my mind as a personal call to action, as I’ve tried to seek out and amplify the work of Black women in West Philadelphia who are doing breastfeeding support. Two years into BAE Cafe, I’m so incredibly thankful for the work that BAE Cafe does to support breastfeeding in my community. Through the unique, trauma-informed model, I know that when I refer my patient’s families to BAE Cafe, they receive so much more than breastfeeding support. They receive advice and partnership that hears their voice and experience and genuinely accompanies them on their journey into parenthood.

**Figure 4.**
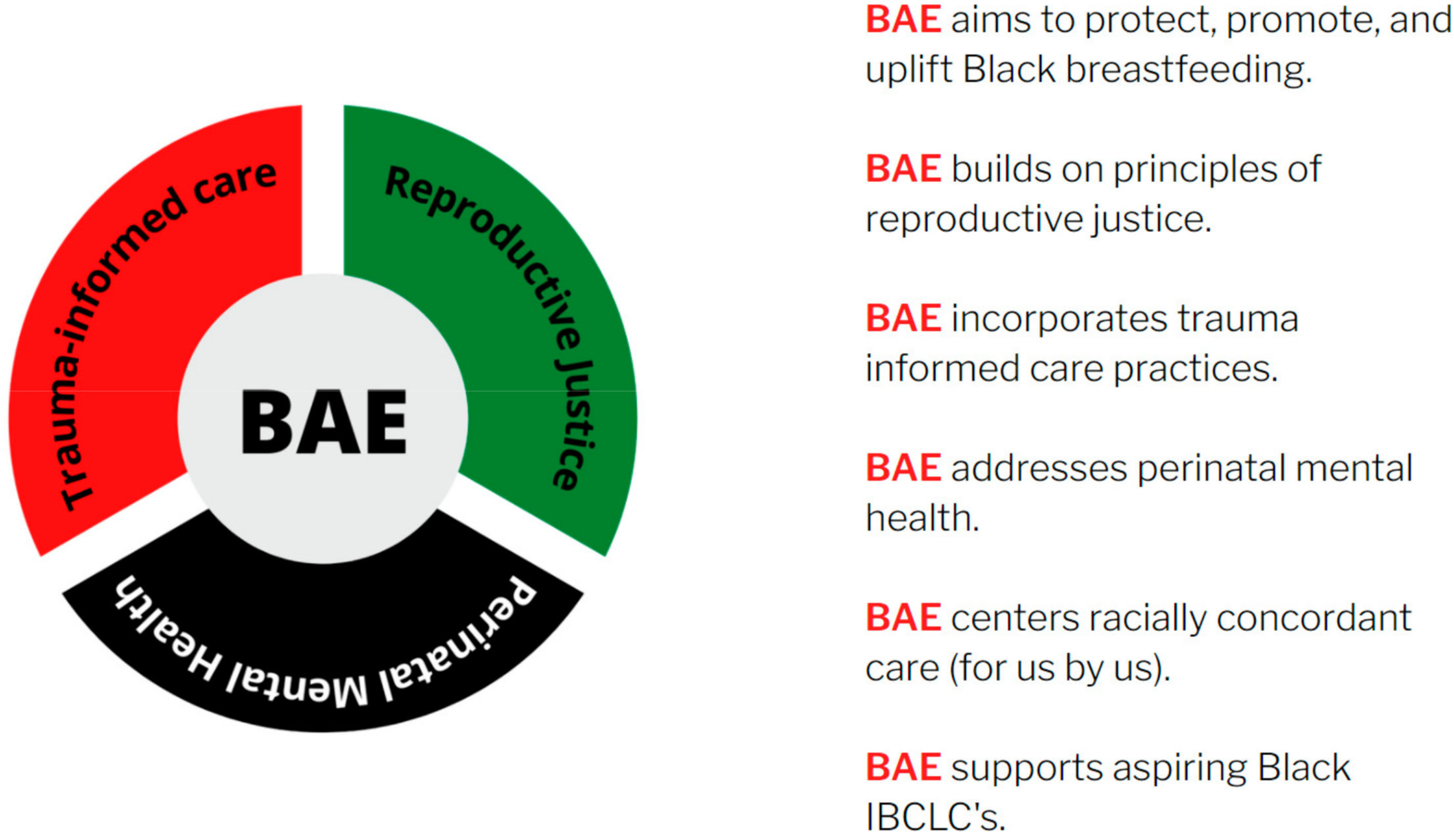
BAE model.

In the survey results, participants specifically addressed relief for a space that felt safe for Black and Brown people (Figure 4). One participant reflected “much of the breastfeeding community and education caters to White families. Love seeing us promote the health and bond of breastfeeding for our children after decades and being robbed of our magic”. Another participant described BAE Cafe as standing out to her as a space “full of support and encouragement from faces of many races with the common goal of being the best parent to their children by making informed decisions with encouraged individuality”.

## 4. Discussion

In this paper, BAE, a Black women-led approach to promoting a multigenerational culture of health, was described to understand the fundamental elements of a program designed to protect, promote and uplift Black breastfeeding. The resulting BAE Cafe program model was then situated in the literature and evaluated through participants’ experience and narrative. The information generated supports a greater understanding of a program model that positively impacts breastfeeding initiation, longevity and perinatal mental health support. As it stands, Black families have decreased access to comprehensive, meaningful lactation support. Nonetheless, we have demonstrated that Black families feel supported in meeting their lactation goals when program models acknowledge the impact of systemic racism on parenting, center their lived experience and uplift the Black breast-feeding experience. The next step in moving the agenda forward to promote health equity and decrease disparities in breastfeeding is to move to action. Based on the conceptual framework of Killborne et al. (2006), Advancing Health Disparities Research Within the Health Care System, we argue that sufficient evidence exists to move from the detection and understanding phases of human milk and lactation health equity work to the reducing phase [46]. In sum, it is time for action. Our brief review of the current literature revealed that while programs have been developed to target breastfeeding initiation and continuation, existing programs do not specifically address race, demonstrate the ability to eliminate health inequity, nor ultimately counter the impact of systemic racism on childbearing families. This reveals a need for strategic program development and implementation that specifically centers Black families.

In addition, the majority (14 out of 16) of the studies were qualitative. While qualitative data do help generate an understanding of the phenomena, they do not move forward the agenda of data collection and analysis of outcomes to track programmatic changes and measure the reduction in target outcomes. Furthermore, in all but one of the studies included, populations were defined as people who identified as low income and/or received public benefits, therefore excluding a broader group of Black people, despite the knowledge that perinatal health inequity transcends income and education levels, leaving the pervasiveness of systemic racism as the prevailing marginalizing factor [11]. The only study that included a broader sample was conducted on social media through support groups, suggesting a potential sample where people from various backgrounds and geographic locations might interact [36]. In addition, no studies included data from partners, family members or support people despite the importance of social network and support. Only one study by Johnson et al. (2016) included information on perinatal mental health, suggesting a gap in the literature and the potential for a further understanding of how and when to integrate perinatal mental health support [29]. Finally, the focus on knowledge gaps about breastfeeding was exhaustive with all studies noting high levels of knowledge of the potential benefits of breastfeeding. This suggests that while it is well understood that most people understand and acknowledge the benefits of breastfeeding, research efforts continue to perpetuate critical gaps by missing meaningful areas of high impact and failing to capture interventions that effectively meet the needs of Black families.

Finally, the literature highlighted multiple support systems and factors that can influence breastfeeding. For example, individual factors, such as family support [33]; network factors, such as social network behaviors [35]; and organizational factors, such clinical bias [44], were all cited. This is consistent with the socio-ecological health behavior model, by which people interact in spheres of influence from the individual to the organizational level [47].

In this context, BAE offers a program model that addresses critical gaps. It also offers a model that includes all Black childbearing families, regardless of income level or education. BAE does not target participants based on income or education, and has included people across a wide spectrum who have shared similar experiences related to transitions to parenting and infant feeding choices. During group observations, these commonalities were apparent, and there was little to no discussion about income level, or professional and financial resources. Rather, participants were engaged in support, sharing and knowledge generation. In the results from the survey, BAE participants responded positively. Over 80% of participants reported having their breastfeeding/chest feeding goals met and reported support of their mental health, and over 75% felt that they had built a community of parental support. Perhaps the strongest evidence that BAE works is that 90% would recommend it to a friend or family member, highlighting its important role in people’s lives during the transition to parenthood. The next steps for BAE include the replication of the BAE Cafe model in a larger population and in other communities at risk, the expansion of the BAE Cafe model to the prenatal population and the dissemination and implementation of the BAE Cafe model for organizations to embed this model into practice. Through these methods, BAE will gain data to strengthen the argument for the model and to support further expansion.

## 5. Conclusions

Resources must be directed toward tailored, comprehensive designs that meet the needs of Black families to combat the critical gaps in the United States healthcare system that fail to address systemic racism and disproportionately perpetuate preventable, adverse reproductive health outcomes for Black people. In this paper, we reviewed the current literature examining interventions for Black families around breastfeeding initiation, continuation and perinatal mental health support. We found that existing literature predominantly explores qualitative anecdotal data and captures outcomes for programs that serve a general population. While information is gained and outcomes moderately improve, very few programs specifically target Black families and effectively offset the impact of systemic racism. It is imperative that we begin to integrate interventions that demonstrate the ability to reduce, and ultimately eliminate, pervasive perinatal health inequity [48]. This must start with program design. Experts write “there is no answer to solving this crisis that Black women do not already know. It is in their lived experiences and resilience that drives innovation and belonging—and we as stakeholders should take heed [49]”. Black people must be at the center of the design and development process for interventions that shape their livelihood. Furthermore, actualization requires full support through comprehensive policies, adequate funding and strategic implementation.

BAE seeks to eliminate disparities for Black childbearing families over time, growth and expansion to mitigate indicated factors influencing reproductive health outcomes. In this paper, we described the key elements of the BAE Cafe model and its grounding in reproductive justice to demonstrate the propriety of this framework. At present, protecting bodily autonomy and honoring people’s decisions around parenting constitute the first step towards moderating outcomes. Second, securing access to safe and healthy choices that promote a stable parent–child connection comes next. Lastly, building strong community relationships, reinforced by shared values and systems of support, amplifies a safety web harnessed by collective power for families to thrive in safe and healthy environments. We present a program model that has demonstrated remarkable success using this framework to implement innovative, evidence-based strategies to increase breastfeeding initiation and continuation among Black families in Philadelphia. Furthermore, participants attest that BAE Cafe has had a positive impact on their perinatal mental and emotional health and strongly influenced their ability to build a supportive community of parents. The presented model should be the standard of care. Only then will we begin to carry out the meaningful work of destabilizing pillars of systemic racism that underline health inequity. With funding support and additional resources, it is our goal to further explore the impact of this model and develop a strategic plan for replication and targeted nationwide dissemination to communities most at risk, advancing BAE’s vision to promote a lasting, multigenerational culture of health.

## Data Availability

This study collected data from programmatic work only and does not use publicly available data.

## Appendix

**Appendix A:**

	BAE Survey
	Qualtrics Survey Questions:
1. What is your year of birth?	
2. What is the highest level of school you have completed or the highest degree you have received?	
a.	Less than high school
b.	High school (GED or diploma)
c.	Some college (no degree)
d.	Associate degree (2 year)
e.	Bachelor’s degree (4 year)
f.	Master’s degree
g.	Doctoral degree
h.	Professional Degree (JD, MD)
3. Are you Spanish, Hispanic, Latino, or none of these?	
a.	Yes
b.	None of these
4. If YES to Question 3: Are you Spanish, Hispanic, or Latino?	
a.	Spanish
b.	Hispanic
c.	Latino
5. Choose one or more races that you consider yourself to be:	
a.	White
b.	Asian
c.	Black/African American
d.	Native Hawaiian or Pacific Islander
e.	American Indian or Alaska Native
f.	Other
6. To which gender do you most identify?	
a.	Female
b.	Male
c.	Gender variant/non-conforming
d.	Transgender male
e.	Transgender female
f.	Not listed______
7. What is your zip code? ________	
8. How many children do you have? ________	
9. Please list the ages of your children. _______	
10. How did you hear about BAE?	
a.	Social media
b.	Provider (Pediatrician, nurse, midwife, lactation consultant or other)
c.	A Friend
d.	Other
11. When did you first come into contact with BAE?	
a.	Pre-Pregnancy
b.	Pregnancy
c.	Postpartum
d.	Other
12. How many BAE Cafe sessions have you attended?	
a.	1
b.	2
c.	3
d.	4
e.	5 or more
13. Why did you initially start breastfeeding?	
14. Are you currently breastfeeding or feeding your child breast milk (some or all)?	
a.	Yes
b.	No
15. If Yes to 14, how long has your child been breastfeeding or fed breast milk (in months)?	
16. If No to 14, at what age did they completely stop breastfeeding or being fed breast milk?	
17. Has your child ever been formula fed?	
a.	Yes
b.	No
18. If YES to 16, how old was your child when they were first formula fed?	
19. How old was your child when they were first fed anything other than breast milk or formula? Please include juice, cow’s milk, sugar water, baby food, or anything that your child may have been given, even water?	
20. How has BAE supported your breastfeeding and parenting journey?	
21. Is there anything about BAE that stands out to you?	
22. Have you referred others to BAE?	
a.	yes
b.	no
23. What would make you refer others to BAE?	
24. What would not make you refer others to BAE?	
25. What is the best way to get the word out about BAE?	
26. On a scale from 0–10 how likely are you to recommend BAE to someone else? 1 2 3 4 5 6 7 8 9 10	
27. Please share any additional comments or feedback regarding BAE in the space provided below?	

## References

[1] CDC. Health objectives for the nation healthy people 2000: National health promotion and disease prevention objectives for the year 2000. MMWR 1990, 5, 695–697. 2119475

[2] Office of the Surgeon General; Centers for Disease Control and Prevention; Office on Women’s Health. The Surgeon General’s Call to Action to Support Breastfeeding; Office of the Surgeon General: Rockville, MD, USA, 2011.

[3] WHO. Guideline: Protecting, Promoting and Supporting Breastfeeding in Facilities Providing Maternity and Newborn Services; World Health Organization: Geneva, Switzerland, 2017. 29565522

[4] Johnston M; Landers S; Noble L; Szucs K; Viehmann L Breastfeeding and the use of human milk. Pediatrics 2012, 3, e827–e841. 10.1542/peds.2011-3552 22371471

[5] Anstey EH; Chen J; Elam-Evans L; Perrine C Racial and geographic differences in breastfeeding—United States, 2011–2015. MMWR Morb. Mortal. Wkly. Rep. 2017, 66, 723–727. [PubMed] 28704352 10.15585/mmwr.mm6627a3 PMC5687589

[6] Beauregard J; Hamner H; Chen J; Avila-Rodriquez W; Elam-Evans L; Perrine C Racial disparities in breastfeeding initiation and duration among U.S. infants born in 2015. Morb. Mortal. Wkly. Rep. 2015, 68, 745. 10.15585/mmwr.mm6834a3 PMC6715261 31465319

[7] City of Philadelphia Community Health Explorer. Available online: https://healthexplorer.phila.gov//racial-disparity (accessed on 11 November 2021).

[8] Breastfeeding Awareness and Empowerment (n.d.). BAE Family. Available online: https://www.baeculture.org/bae-family (accessed on 13 October 2021).

[9] Muse S Setting the Standard for Holistic Care of and for Black Women; Black Mamas Matter Alliance: Atlanta, GA, USA, 2018.

[10] Scott KA; Britton L; McLemore MR The ethics of perinatal care for black women: Dismantling the structural racism in “mother blame” narratives. J. Perinat. Neonatal. Nurs. 2019, 33, 108–115. 31021935 10.1097/JPN.0000000000000394

[11] Joseph J We Cannot Save Black Women in America if We Don’t Start Telling the Truth; Black Maternal Health Caucus: Washington, DC, USA, 2019.

[12] Chiang KV; Li R; Anstey EH; Perinne CG Racial and ethnic disparities in breastfeeding initiation—United States. Morb. Mortal. Wkly. Rep. 2021, 70, 769–774. 10.15585/mmwr.mm7021a1 PMC8158892 34043611

[13] Stuebe A #BlackLivesMatter and breastfeeding medicine: A call to action. Breastfeed. Med. 2020, 15, 479–480. 32614243 10.1089/bfm.2020.29158.ams

[14] Jones KM; Power ML; Queenan JT; Schulkin J Racial and ethnic disparities in breastfeeding. Breastfeed. Med. 2015, 5, 186–196. 10.1089/bfm.2014.0152 PMC4410446 25831234

[15] Robinson K; Fial A; Hanson L Racism, bias, and discrimination as modifiable barriers to breastfeeding for african american women: A scoping review of the literature. J. Midwifery Women’s Health 2019, 11, 734–742. 10.1111/jmwh.13058 31710173

[16] United States Lactation Consultant Association. 2019 Lactation Care Provider Demographic Survey. Report No. 2020–06–08T20, 13, 08+00, 00. Available online: https://uslca.org/wp-content/uploads/2020/06/2019-Lactation-Care-Provider-Demographic-Survey.pdf (accessed on 9 October 2021).

[17] Griswold MK; Crawford SL; Perry DJ; Person SD; Rosenberg L; Cozier YC; Palmer JR Experiences of racism and breastfeeding initiation and duration among first-time mothers of the black women’s health study. J. Racial Ethn. Health Disparities 2018, 12, 1180–1191. 10.1007/s40615-018-0465-2 PMC6681652 29435898

[18] Street RL; O’Malley KJ; Cooper LA; Haidet P Understanding concordance in patient-physician relationships: Personal and ethnic dimensions of shared identity. Ann. Fam. Med. 2008, 6, 198–205. 18474881 10.1370/afm.821 PMC2384992

[19] Julian Z; Robles D; Whetstone S; Perritt JB; Jackson AV; Hardeman RR; Scott KA Community-informed models of perinatal and reproductive health services provision: A justice-centered paradigm toward equity among black birthing communities. Semin. Perinatol. 2020, 44, 151267. 32684310 10.1016/j.semperi.2020.151267

[20] Sperlich M; Seng JS; Li Y; Taylor J; Bradbury-Jones C Integrating trauma-informed care into maternity care practice: Conceptual and practical issues. J. Midwifery Women’s Health 2017, 11, 661–672. 10.1111/jmwh.12674 29193613

[21] Honikman JI The role of Postpartum support international in helping perinatal families. J. Obstet. Gynecol. Neonatal. Nurs. 2006, 35, 659–661. 10.1111/j.1552-6909.2006.00088.x 16958724

[22] Ethnic/Racial Diversity, Maternal Stress, Lactation and Very Low Birthweight Infants: DigitalCommons@TMC. 2007. Available online: https://digitalcommons.library.tmc.edu/baylor_docs/8 (accessed on 14 November 2021).

[23] Reproductive Justice. Available online: https://www.sistersong.net/reproductive-justice (accessed on 14 November 2021).

[24] Infographic: 6 Guiding Principles to a Trauma-Informed Approach. CDC. 2020. Available online: https://www.cdc.gov/cpr/infographics/6_principles_trauma_info.htm (accessed on 14 November 2021).

[25] Wouk K; Stuebe A; Meltzer-Brody S Postpartum mental health and breastfeeding practices: An analysis using the 2010–2011 pregnancy risk assessment monitoring system. Matern. Child Health J. 2017, 7, 636–647. 10.1007/s10995-016-2150-6 PMC5253344 27449655

[26] Black Breastfeeding Week (n.d.). Available online: https://blackbreastfeedingweek.org (accessed on 15 November 2021).

[27] Hammond W Principles of strength-based practice. Resiliency Initiat. 2010, 12, 1–7.

[28] Kadakia A; Joyner B; Tender J; Oden R; Moon RY Breastfeeding in African Americans may not depend on sleep arrangement: A mixed-methods study. Clin. Pediatr. 2015, 54, 47–53. 10.1177/0009922814547565 PMC4377646 25139664

[29] Johnson AM; Kirk R; Rooks AJ; Muzik M Enhancing breastfeeding through healthcare support: Results from a focus group study of African american mothers. Matern. Child Health J. 2016, 20, 92–102. 27449776 10.1007/s10995-016-2085-y PMC5290044

[30] Lewkowitz AK; López JD; Stein RI; Rhoades JS; Schulz RC; Woolfolk CL; Macones GA; Haire-Joshu D; Cahill AG Effect of a home-based lifestyle intervention on breastfeeding initiation among socioeconomically disadvantaged African American women with overweight or obesity. Breastfeed. Med. 2018, 13, 418–425. 29912571 10.1089/bfm.2018.0006 PMC6065521

[31] CDC. (2021, November 1). Breastfeeding.. Available online: http://www.cdc.gov/breastfeeding (accessed on 27 October 2021).

[32] Carlin RF; Mathews A; Oden R; Moon RY The influence of social networks and norms on breastfeeding in African American and Caucasian mothers: A qualitative study. Breastfeed. Med. 2019, 14, 640–647. 31433206 10.1089/bfm.2019.0044 PMC6857545

[33] Pugh LC; Serwint JR; Frick KD; Nanda JP; Sharps PW; Spatz DL; Milligan RA A randomized controlled community-based trial to improve breastfeeding rates among urban low-income mothers. Acad. Pediatr. 2010, 10, 14–20. 19854119 10.1016/j.acap.2009.07.005 PMC2818063

[34] Gross SM; Caulfield LE; Bentley ME; Bronner Y; Kessler L; Jensen J; Paige DM Counseling and motivational videotapes increase duration of breast-feeding in African-American WIC participants who initiate breast-feeding. J. Am. Diet Assoc. 1998, 98, 143–148. 12515413 10.1016/s0002-8223(98)00037-6

[35] Reno R Using group model building to develop a culturally grounded model of breastfeeding for low-income African American women in the USA. J. Clin. Nurs. 2018, 27, 3363–3376. 28252834 10.1111/jocn.13791

[36] Robinson A; Davis M; Hall J; Lauckner C; Anderson AK It takes an e-village: Supporting African American mothers in sustaining breastfeeding through facebook communities. J. Hum. Lact. 2019, 35, 569–582. 30889373 10.1177/0890334419831652

[37] Sutton MY; Anachebe NF; Lee R; Skanes H Racial and ethnic disparities in reproductive health services and outcomes, 2020. Obstet. Gynecol. 2021, 137, 225–233. 33416284 10.1097/AOG.0000000000004224 PMC7813444

[38] Lutenbacher M; Karp SM; Moore ER Reflections of black women who choose to breastfeed: Influences, challenges and supports. Matern. Child Health J. 2016, 20, 231–239. 26496988 10.1007/s10995-015-1822-y

[39] Johnson AM; Kirk R; Muzik M Overcoming workplace barriers: A focus group study exploring African American mothers’ needs for workplace breastfeeding support. J. Hum. Lact. 2015, 31, 425–433. 25714345 10.1177/0890334415573001 PMC4506723

[40] Obeng CS; Emetu RE; Curtis TJ African-American women’s perceptions and experiences about breastfeeding. Front. Public Health 2015, 3, 273. 26734597 10.3389/fpubh.2015.00273 PMC4685054

[41] Cottrell BH; Detman LA Breastfeeding concerns and experiences of African American mothers. MCN Am. J. Matern. Child Nurs. 2013, 38, 297–304. 23958620 10.1097/NMC.0b013e31829a5606

[42] Fabiyi C; Peacock N; Hebert-Beirne J; Handler A A qualitative study to understand nativity differences in breastfeeding behaviors among middle-class African American and African-born women. Matern. Child Health J. 2016, 20, 2100–2111. 27334637 10.1007/s10995-016-2029-6

[43] Lewallen LP; Street DJ Initiating and sustaining breastfeeding in african american women. J. Obstet. Gynecol. Neonatal Nurs. 2010, 39, 667–674. [PubMed] 10.1111/j.1552-6909.2010.01196.x 21044149

[44] Fischer TP; Olson BH A qualitative study to understand cultural factors affecting a mother’s decision to breast or formula feed. J. Hum. Lact. 2014, 30, 209–216. [PubMed] 24186645 10.1177/0890334413508338

[45] Ware JL; Love D; Ladipo J; Paddy K; Starr M; Gilliam J; Miles N; Leatherwood S; Reese L; Baker T African American breastfeeding peer support: All moms empowered to nurse. Breastfeed. Med. 2021, 6, 156–164. [PubMed] 10.1089/bfm.2020.0323 PMC8020535 33591227

[46] Kilbourne AM; Switzer G; Hyman K; Crowley-Matoka M; Fine MJ Advancing health disparities research within the health care system: A conceptual framework. Am. J. Public Health 2006, 96, 2113–2121. 17077411 10.2105/AJPH.2005.077628 PMC1698151

[47] Snyder K; Hulse E; Dingman H; Cantrell A; Hanson C; Dinkel D Examining supports and barriers to breastfeeding through a socio-ecological lens: A qualitative study. Int. Breastfeed. J. 2021, 16, 52. 34247633 10.1186/s13006-021-00401-4 PMC8273968

[48] National Academies of Sciences, Engineering, Division HaMPractice, Board on Population Health and Public Health States, Committee on Community-Based Solutions to Promote Health Equity in the UnitedBaciu, A.; Negussie Y The Root Causes of Health Inequity; National Academies Press: Washington, DC, USA, 2017.

[49] An Inconvenient Truth: You Have No Answer that Black Women Don’t Already Possess. 2018. Available online: https://precisionmedicine.ucsf.edu/publications/inconvenient-truth-you-have-no-answer-black-women-don\T1\textquoterightt-already-possess-0 (accessed on 14 November 2021).

